# Rituximab may affect T lymphocyte subsets balance in primary membranous nephropathy

**DOI:** 10.1186/s12882-024-03521-1

**Published:** 2024-03-06

**Authors:** Yuanyuan Zhang, Jingjing Yang, Jianzhong Li, Jiani Sun, Ling Zhou, Deyu Xu, Wengang Sha, Lan Dai, Lei Shen

**Affiliations:** 1 Department of Nephrology, The First Affiliated Hospital of Soochow University, Suzhou, PR China; 2 Department of Nephrology, Suzhou Hospital, Affiliated Hospital of Medical School, Nanjing University, Suzhou, PR China; 3 Department of Nephrology, BenQ Medical Center, Suzhou, PR China; 4 Jiangsu Institute of Hematology, Key Laboratory of Thrombosis and Hemostasis of Ministry of Health, The First Affiliated Hospital of Soochow University, Suzhou, PR China

**Keywords:** Rituximab, Primary membranous nephropathy, T lymphocyte subsets, B lymphocyte cells, Anti-PLA2R antibody

## Abstract

**Background:**

The aim of this study was to investigate the effects and significance of rituximab (RTX) on the levels of T lymphocyte subsets in patients diagnosed with primary membranous nephropathy (PMN).

**Methods:**

A total of 58 PMN patients and 25 healthy donors were chosen as the subjects. Among the PMN patients, 40 individuals received RTX treatment and completed at least 6 months of follow-up. All subjects underwent flow cytometry analysis to determine the peripheral blood lymphocyte subsets. The changes in anti-PLA2R antibody titers and 24-hour urinary protein levels were evaluated by ELISA and Biuret method before and after treatment.

**Results:**

(1) The PMN group exhibited a significantly greater percentage of peripheral blood CD3^−^CD19^+^ B cells than the healthy group, which is consistent with the findings of previous reports. Additionally, compared with those in the peripheral blood of healthy individuals, the numbers of CD4^+^ central memory T cells, CD4^+^ effector memory T cells, CD4^+^/CD8^+^, and CD4^+^CD25^+^ T cells in the PMN peripheral blood were markedly greater. However, the number of peripheral blood Treg cells was reduced in the PMN group. (2) After 6 months of RTX treatment, PMN patients exhibited significant decreases in anti-PLA2R antibody titers, 24-hour urinary protein levels, and peripheral blood CD3^−^CD19^+^ B cells. Importantly, RTX administration decreased CD4^+^CD25^+^ T cells and CD4^+^/CD8^+^ in the peripheral blood of PMN patients and improved Treg cell levels. (3) RTX treatment induced alterations in the CD4^+^ T lymphocyte subsets in PMN patients, which did not correlate with B lymphocyte counts or anti-PLA2R antibody titers.

**Conclusions:**

RTX treatment might have a beneficial impact on cellular immunity by effectively restoring the balance of CD4^+^ T lymphocyte subsets in PMN patients, which is beyond its effects on B cells and antibody production.

**Trial registration:**

The research was registered at the First Affiliated Hospital of Soochow University. Registration Number: MR-32-23-016211. Registration Date: May 31, 2023.

**Supplementary Information:**

The online version contains supplementary material available at 10.1186/s12882-024-03521-1.

## Introduction

Primary membranous nephropathy (PMN) is the most common cause of nephrotic syndrome in adults, accounting for approximately 25-40% of cases of primary nephrotic syndrome, and is a leading cause of end-stage renal disease (ESRD) [1–2]. PMN is now recognized as an autoimmune disease caused by the discovery of specific antigens such as the anti-phospholipase A2 receptor (PLA2R) antibody and thrombospondin type-1 domain-containing 7 A (THSD7A) [3–5]. Recently, rituximab (RTX), a chimeric monoclonal antibody that targets the CD20 cell surface receptor expressed on B cells, has emerged as a therapeutic agent for PMN. RTX binds specifically to B cells, inducing their apoptosis and reducing plasma cell secretion of antibodies such as anti-PLA2R [6]. The 2021 KIDIGO guidelines now recommend RTX as a first-line treatment for PMN patients [7]. However, approximately 35-40% of PMN patients do not respond clinically to RTX and often progress to ESRD within 10–15 years [8]. Therefore, further investigation into the therapeutic mechanism underlying the role of RTX in the PMN is crucial.

While B-cell depletion and a reduction in disease-causing antibodies are believed to be the primary therapeutic mechanisms of RTX, we have observed sustained clinical remission in PMN patients with incomplete B-cell depletion during early-stage RTX treatment or with B-cell recovery in the late stage of treatment. Additionally, RTX has demonstrated therapeutic efficacy in diseases primarily associated with T lymphocyte dysfunction, such as minimal change nephropathy and focal segmental glomerulonephritis [9–10].

T lymphocyte subsets are mainly divided into CD4 + helper T cells, CD8 + cytotoxic T cells, and CD3 + CD4-CD8- double negative T cells according to the differences in the surface differentiation antigen CD molecules [11]. CD4 + T cells play an essential role in humoral and cellular immunity and are related to the degree of the body’s immune system activity. CD8 + T cells can inhibit pathogenic CD4 + T cells in autoimmune diseases and kill target cells [12]. CD4 + T cells can be divided into CD4 + central memory T cells, CD4 + effector memory T cells and regulatory T cells according to phenotype. CD4 + central memory T cells have a high diversity of T-cell receptors, can respond to various antigens, and play an important role in protecting the body from infection. CD4 + effector memory T cells can rapidly produce many effector molecules, such as cytokines and inflammatory mediators, for the immune response [13]. The regulatory T cells (Tregs) we focused on are a special class of T lymphocytes that are CD4 + T cells. In addition to effectively inhibiting the autoimmune response, Tregs may also be involved in inducing transplantation tolerance and regulating tumour immunity and play a key role in maintaining the stability of the body’s internal environment [14]. Previous studies have shown that glomerulonephritis can cause immune dysfunction through an imbalance of T lymphocyte subsets [15]. Several studies have shown an increase in the proportion of CD4^+^/CD8^+^ T cells in PMN patients, with the CD4^+^/CD8^+^ ratio gradually decreasing with the clinical remission of nephropathy [16–17]. Clinical trials conducted by Ronco et al., primarily involving RTX, have also indicated reduced Treg cell counts and disrupted immune tolerance in PMN patients [18–20]. Therefore, whether RTX affects T lymphocytes and its impact on PMN must be investigated.

In this study, we identified T lymphocyte subsets exhibiting abnormal alterations and determined whether this could be rectified following RTX treatment in the PMN. Furthermore, we aimed to evaluate the clinical significance of T lymphocyte subsets in the response to RTX treatment, thereby establishing a foundation for a further comprehensive evaluation of the role of RTX in PMN.

## Materials and methods

### Participants

A total of 58 PMN patients admitted to the Department of Nephrology, the First Affiliated Hospital of Soochow University, from August 2021 to August 2023 were selected as the research subjects. Among the PMN patients, 40 individuals received RTX treatment and completed at least 6 months of follow-up.

The inclusion criteria for patients were as follows: (a) PMN diagnosed by renal biopsy; (b) aged 18–75 years; and (c) had complete baseline data, such as peripheral blood anti-PLA2R antibody titer and urine protein quantification.

The exclusion criteria for patients were as follows: (a) secondary membranous nephropathy caused by autoimmune diseases (such as hepatitis B virus infection, systemic lupus erythematosus, rheumatoid arthritis, etc.), malignant tumours, infections, drugs, etc.; (b) combined malignant tumours, blood system diseases, decompensated liver cirrhosis, heart failure, serious infections such as lung infection, peritonitis; (c) current active infection or combined with severe immunodeficiency diseases; (d) pregnant or (and) lactation; (e) rapidly progressive nephritis with severe renal impairment (eGFR < 15 ml/min/1.73 m^2^).

Twenty-five healthy age- and sex-matched people with an eGFR ≥ 60 ml/min/1.73 m^2^ and a negative urinary protein level who underwent physical examination at the health management centre of our hospital in 2021 were randomly selected as the healthy control group.

### Methods

Flow cytometry was used to analyse the distribution of lymphocytes. Two millilitres of venous blood was collected from the two groups, stored at room temperature for 20 min and centrifuged at low speed (speed: 3500 r/min; radius: 8 cm). After 10 min, the upper serum sample was collected, ammonium chloride was used as the haemolysis agent, and then PBS (pH = 7.4, containing 0.5% BSA) was added after haemolysis. We centrifuged the cells twice every 5 min, after which the supernatant was discarded. Two tubes of 1 × 10^6^ cells were added to two tubes of CDS dry powder for immune function and incubated at room temperature in the dark for 30 min. Then, we added PBS, centrifuged the cells, and washed them once every 5 min. The supernatant was discarded, and 500 µl of PBS (pH = 7.4, 0.5% PFA) was added to resuspend the cells. Flow cytometry was used to determine the distribution of peripheral blood lymphocytes, which included CD3^+^ T lymphocytes, CD3^+^CD4^+^ T cells, CD3^+^CD8^+^ T cells, mature NK cells (CD16^+^CD56^+^), B lymphocytes (CD3^−^CD19^+^), DNT cells (CD3^+^ CD4^−^CD8^−^), Treg cells (CD4^+^CD25^+^CD127^lo^), and CD4^+^CD25^+^ T cells. The kit used in this study was obtained from the German company Partec, and the testing instrument used was a CyFlowCube8 flow cytometer produced by the German company Partec. Finally, Kaluza analysis software was used to calculate the percentage of each lymphocyte subgroup among the peripheral blood lymphocytes based on the peripheral blood lymphocyte gates. The results of anti-PLA2R antibody titre and 24-hour urinary protein quantification were collected for all subjects before and after treatment.

### Rituximab treatment regimen

Rituximab (Shanghai Fuhong Hanlin Biopharmaceutical Co., Ltd., National Drug Approval Number: S20190021, specification: 500 mg/50 ml) was injected by intravenous drip according to the following regimen:

Regimen 1: Standard-dose RTX 375 mg/m^2^ body surface area (BSA) was administered intravenously once every 2 weeks for a total of 4 doses.

Regimen 2: Standard dose of RTX (1 g/time, once every two weeks, intravenously, twice in total).

Regimen 3: Nonstandard dose of RTX 100 mg/time, once a week, 3 times in total.

### Statistical methods

Continuous variables were expressed as the mean ± standard deviation or median (quartile), and categorical variables were expressed as the number of patients (percentage). Independent sample t tests, nonparametric tests or chi-square tests were used to compare the differences between the data between groups. Correlations among variables were analysed by Spearman correlation. *P* < 0.05 was considered to indicate statistical significance, and the statistical analysis of the data was carried out with Microsoft version SPSS 23.0.

## Results

### Trial participants

A total of 58 PMN patients were selected for this study; 41 were males (71%), 17 were females (29%), and the median baseline age was 53 years. There were 25 healthy controls, including 14 males (56%) and 11 females (44%), with a median age of 47 years old. There was no significant difference in sex or age between the two groups (*P* > 0.05; Supplementary Table 1). Compared with healthy individuals, PMN patients had lower albumin levels and higher triglyceride and cholesterol levels. EGFR and serum creatinine levels were normal in both groups, although there were significant differences between the two groups (Supplementary Table 1). Forty PMN patients (27 males and 13 females) were assigned to one of three RTX intervention regimen trial groups according to the KDIGO guidelines and clinical experience, and 100% completed the treatment period and at least 6 months of follow-up. The 40 patients were followed up for an average of 14.5 months after RTX treatment.

### Disorder of T-cell subsets in the peripheral blood of PMN patients

**Fig. 1:**
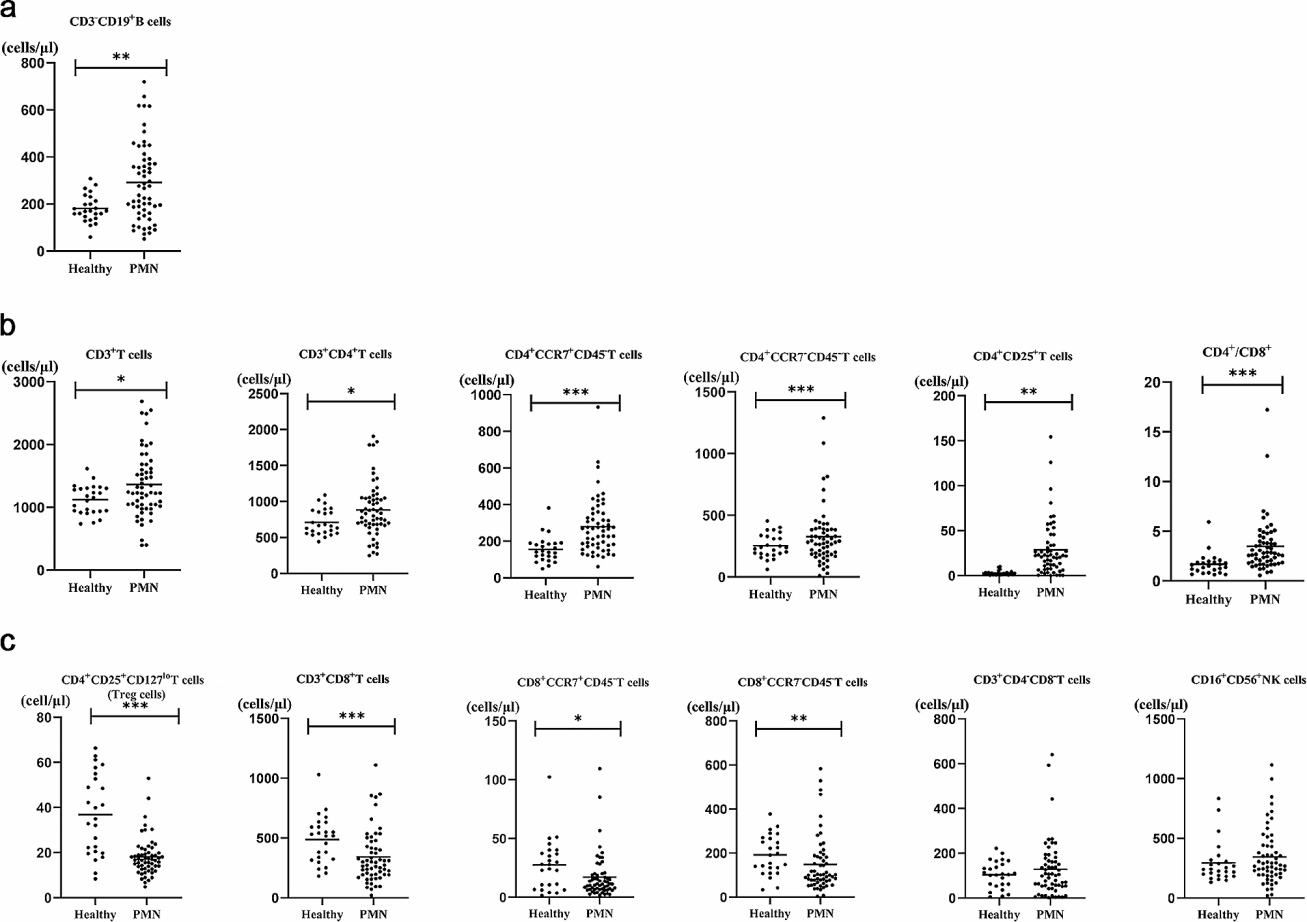
Comparison of B lymphocyte, NK cell and T lymphocyte subsets in the PMN group and healthy group (*P < 0.05; **P < 0.01; ***P < 0.001)

First, we measured the level of B cells in the peripheral blood of PMN patients and healthy participants. As expected, the number of CD3^−^CD19^+^ B cells was markedly greater in the PMN group (*P* = 0.004; Fig. 1a). Notably, PMN patients exhibited an imbalance in T-cell subsets, mainly including an increase in CD4^+^ T-cell subsets and a decrease in CD8^+^ T-cell subsets. As shown in Fig. 1b, the numbers of CD3^+^ T cells (*P* = 0.048), CD3^+^CD4^+^ T cells (*P* = 0.017), CD4^+^ central memory T cells (CD4^+^CCR7^+^CD45RA^−^) (*P* < 0.001), and CD4^+^ effector memory T cells (CD4^+^CCR7^−^CD45RA^−^) (*P* < 0.001), and of CD4^+^CD25^+^ T cells (IL-2Rα) (*P* < 0.001) and CD4^+^/CD8^+^ (*P* < 0.001) were significantly greater in PMN patients than in healthy controls (Fig. 1b). However, the percentage of Treg cells (CD4^+^CD25^+^CD127^lo^) in the PMN group was lower (*P* < 0.001) (Fig. 1c). Additionally, CD3^+^CD8^+^T cells (*P* = 0.001), CD8^+^ central memory T cells (CD8^+^CCR7^+^CD45RA^−^) (*P* = 0.022) and CD8^+^ effector memory T cells (CD8^+^CCR7^−^CD45RA^−^) (*P* = 0.007) were obviously reduced in the PMN group. There was no statistically significant difference in double-negative T cells (CD3^+^CD4^−^CD8^−^) or mature NK cells (CD16^+^CD56^+^) between the two groups (*P* > 0.05) (Fig. 1c). The above detailed data are shown in Table 1.

**Table 1:** Comparison of cellular immune indices between the PMN group and the healthy group

	Healthy Group (*n* = 25)	PMN Group (*n* = 58)	Z	P
**CD3** ^**+**^ **T, cells/µl**	1025.29(784.13, 1293.04)	1234.04(1019.45, 1635.95)	-1.975	0.048
**CD3** ^**+**^ **CD4** ^**+**^ **T, cells/µl**	659.25(558.13, 867.24)	836.30(679.06, 1049.29)	-2.392	0.017
**CD4** ^**+**^ **central memory T, cells/µl**	141.39(107.10, 188.80)	261.62(176.00, 351.60)	-4.298	< 0.001
**CD4** ^**+**^ **effect memory T, cells/µl**	231.51(201.07, 268.57)	290.32(187.13, 388.35)	-4.729	< 0.001
**CD4** ^**+**^ **CD25** ^**+**^ **T, cells/µl**	2.44(1.12, 3.39)	21.91(7.73, 34.79)	-5.499	< 0.001
**CD4** ^**+**^ **CD25** ^**+**^ **CD127** ^**lo**^ **T (Treg), cells/µl**	37.74(22.10, 61.15)	18.67(10.68, 34.35)	-3.861	< 0.001
**CD3** ^**+**^ **CD8** ^**+**^ **T, cells/µl**	517.92(320.26, 612.81)	285.55(195.48, 423.27)	-3.325	0.001
**CD8** ^**+**^ **central memory T, cells/µl**	27.87(8.57, 38.69)	11.14(6.66, 19.19)	-2.293	0.022
**CD8** ^**+**^ **effect memory T, cells/µl**	191.30(121.39, 268.35)	100.73(71.39, 184.87)	-2.710	0.007
**CD8** ^**+**^ **CD25** ^**+**^ **T, cells/µl**	1.49(0.28, 7.16)	1.43(0.28, 7.88)	-0.178	0.858
**CD4** ^**+**^ **/CD8** ^**+**^	1.46(1.06, 2.39)	2.73(1.84, 4.61)	-4.065	< 0.001
**CD3** ^**+**^ **CD4** ^**-**^ **CD8** ^**-**^ **T, cells/µl**	104.70(57.63, 144.02)	103.17(53.39, 168.13)	-0.238	0.812
**CD16** ^**+**^ **CD56** ^**+**^ **NK, cells/µl**	201.87(160.84, 289.20)	238.21(195.31, 438.86)	-1.122	0.262

PMN, Primary membranous nephropathy

### RTX treatment reduced B-cell and anti-PLA2R titres in PMN patients

**Fig. 2:**
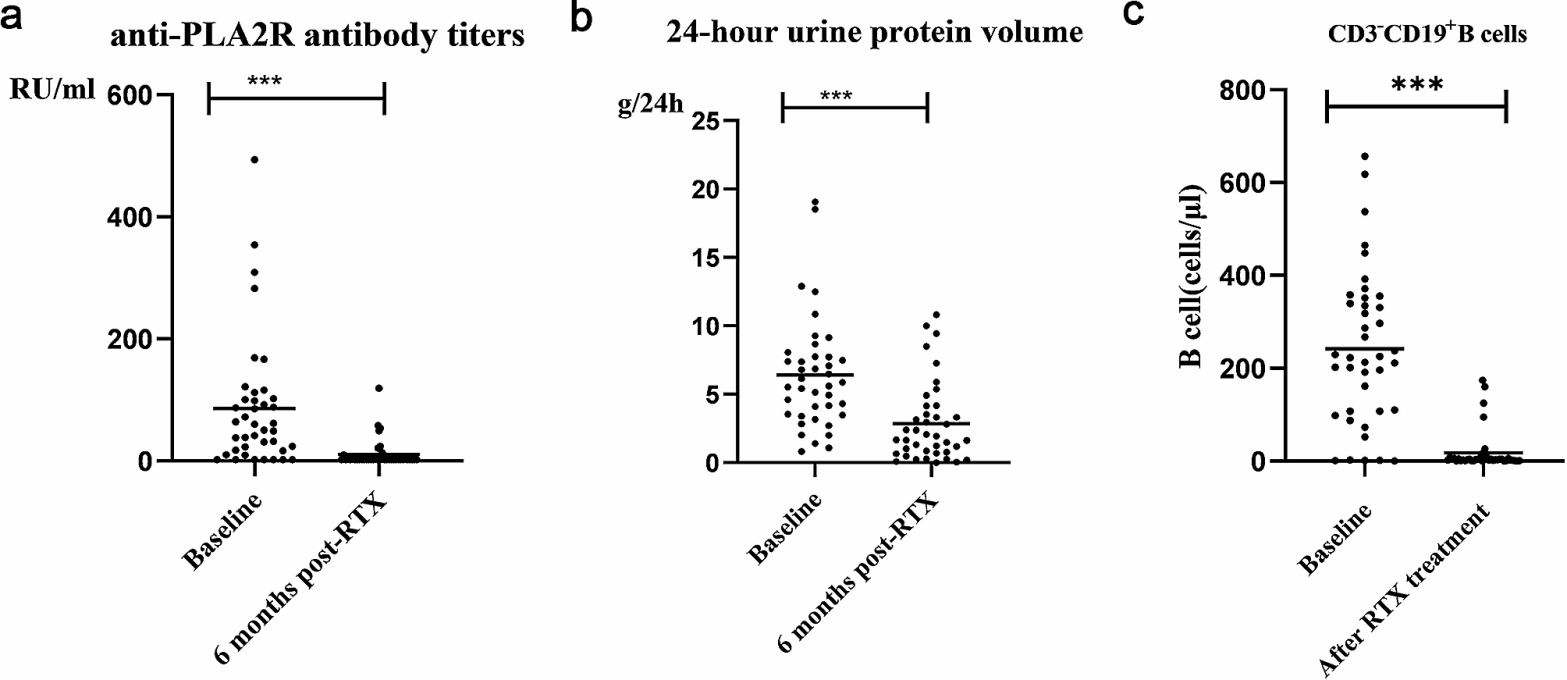
Changes in clinical indices and B lymphocytes after RTX treatment (**P < 0.01; ***P < 0.001)

Forty PMN patients were treated with RTX and followed up for at least 6 months; 26 patients were treated with regimen 1, 8 patients with regimen 2, and 6 patients with regimen 3. During treatment, 5 patients experienced adverse events after medication, including 1 case of allergy and 4 cases of upper respiratory tract infection. During the follow-up, 7 patients achieved complete remission after treatment, 22 patients experienced partial remission, and 11 patients experienced no remission or relapse after remission. A statistical analysis indicated an improvement in clinical indicators in PMN patients after RTX treatment, as indicated by an obvious reduction in as indicated by an obvious reduction in anti-PLA2R antibody titres (Fig. 2a) and 24-hour urine protein volume (Fig. 2b). Additionally, flow cytometry results showed that CD3-CD19 + B-cell numbers were markedly decreased in PMN patients after RTX administration (Fig. 2c). These results further confirm the RTX-mediated therapeutic effect on PMNs, which is consistent with the findings of previous reports.

### RTX treatment improved T lymphocyte subsets imbalance in PMN patients

**Fig. 3:**
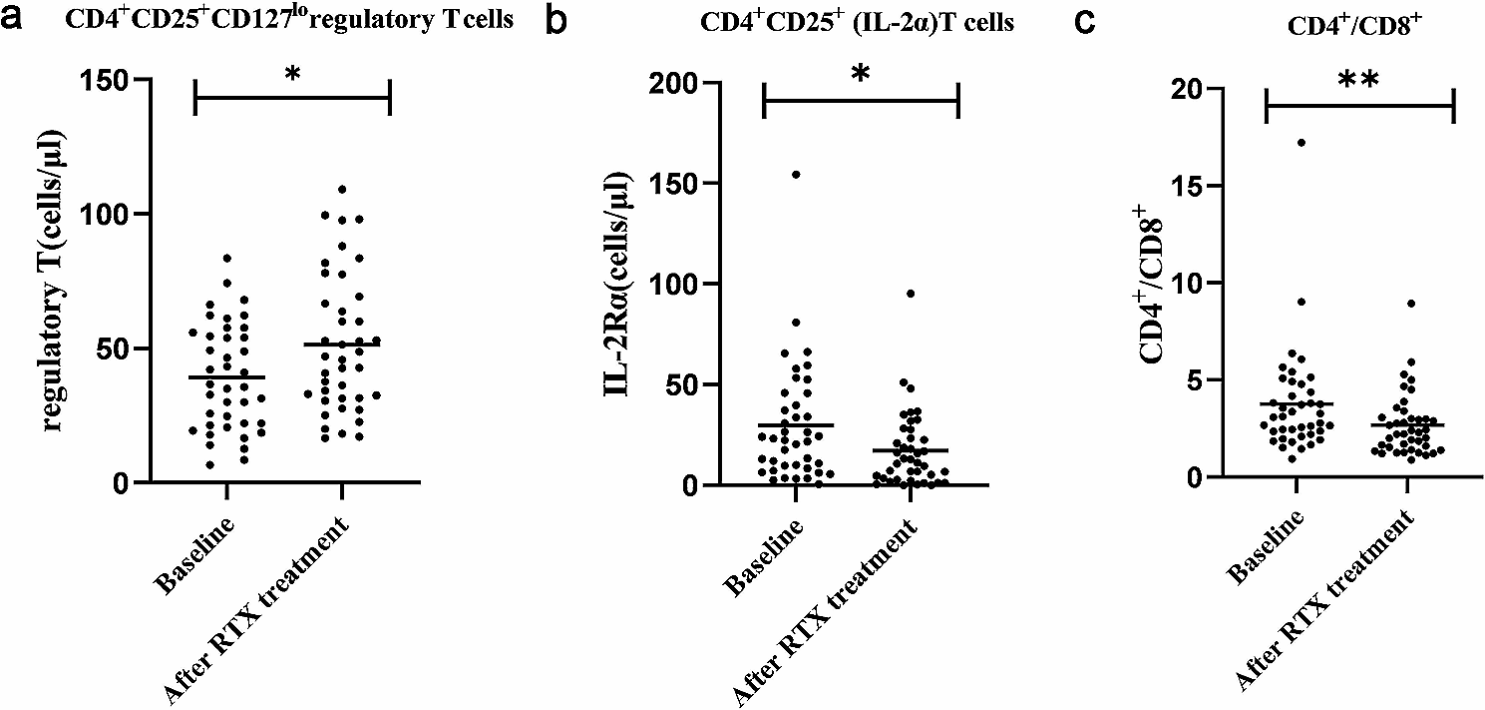
Distribution of T lymphocyte subsets after RTX treatment (*P < 0.05; **P < 0.01)

The total T lymphocyte count was not altered when RTX was used (Table 2). We further explored the changes in T-cell subsets after RTX treatment. As shown in Fig. 3a, the percentage of peripheral blood Treg cells (CD4^+^CD25^+^CD127^lo^) in PMN patients was significantly elevated after 6 months of RTX treatment (*P* = 0.019). In comparison, RTX treatment corrected some of the disordered CD4^+^ T cells described above, including CD4^+^CD25^+^ T lymphocytes (*P* = 0.016) (Fig. 3b) and CD4^+^/CD8^+^ levels (Fig. 3c) were decreased (*P* = 0.002). Moreover, the numbers of CD3^+^ T cells, CD4^+^ T cells, CD4^+^ central memory T cells (CD4^+^CCR7^+^CD45RA^−^), CD4^+^ effector memory T cells (CD4^+^CCR7^−^CD45RA^−^), CD3^+^CD8^+^ T cells, CD8^+^ central memory T cells (CD8^+^CCR7^+^CD45RA^−^), and CD8^+^ effector memory T cells (CD8^+^CCR7^−^CD45Ra^−^), as well as double-negative T cells (CD3^+^CD4^−^CD8^−^), and NK cell counts were not affected by RTX (*P* > 0.05). The above detailed data are shown in Table 2.

**Table 2:** Changes in cellular immune indicators in the PMN patients before and after RTX treatment

	Before treatment (*n* = 40)	6 months after RTX (*n* = 40)	T/Z	P
**CD3** ^**+**^ **T, cells/µl**	1239.61(1051.61, 1549.25)	1219.13(964.04, 1497.86)	-1.438	0.150
**CD3** ^**+**^ **CD4** ^**+**^ **T, cells/µl**	851.43(695.34, 1065.91)	793.47(631.40, 977.24)	-1.317	0.188
**CD4** ^**+**^ **central memory T, cells/µl**	234.40(16.10, 351.96)	192.21(147.13, 284.50)	-1.317	0.188
**CD4** ^**+**^ **effect memory T, cells/µl**	288.19(201.33, 391.48)	301.06(232.56, 364.90)	-0.430	0.667
**CD4** ^**+**^ **CD25** ^**+**^ **T, cells/µl**	22.82(8.74, 44.30)	12.07(2.90, 26.64)	-2.419	0.016
**CD4** ^**+**^ **CD25** ^**+**^ **CD127** ^**lo**^ **T (Treg), cells/µl**	36.15(21.59, 55.58)	46.46(31.44, 68.63)	-2.352	0.019
**CD3** ^**+**^ **CD8** ^**+**^ **T, cells/µl**	256.93(191.14, 335.21)	283.47(202.70, 515.05)	-1.828	0.068
**CD8** ^**+**^ **central memory T, cells/µl**	9.60(5.87, 15.92)	11.52(5.55, 18.90)	-0.659	0.510
**CD8** ^**+**^ **effect memory T, cells/µl**	93.59(76.32, 174.86)	114.75(73.03, 91.00)	-0.301	0.763
**CD8** ^**+**^ **CD25** ^**+**^ **T, cells/µl**	0.87(0.08, 4.03)	1.43(0.30, 7.44)	-2.050	0.040
**CD4** ^**+**^ **/CD8** ^**+**^	3.20(2.36, 5.05)	2.80(1.91, 3.60)	-3.051	0.002
**CD3** ^**+**^ **CD4** ^**-**^ **CD8** ^**-**^ **T, cells/µl**	109.43(54.89, 165.58)	12.27(4.59, 102.58)	-1.507	0.132
**CD16** ^**+**^ **CD56** ^**+**^ **NK, cells/µl**	243.41(201.33, 453.88)	317.06(175.63, 490.22)	-1.129	0.259
**CD3** ^**-**^ **CD19** ^**+**^ **B, cells/µl**	268.30(179.58, 371.47)	11.89(1.60, 102.39)	-5.256	< 0.001

PMN, Primary membranous nephropathy; RTX, rituximab

### T-cell subsets changes were not correlated with B-cell or anti-PLA2R antibody titres

**Fig. 4:**
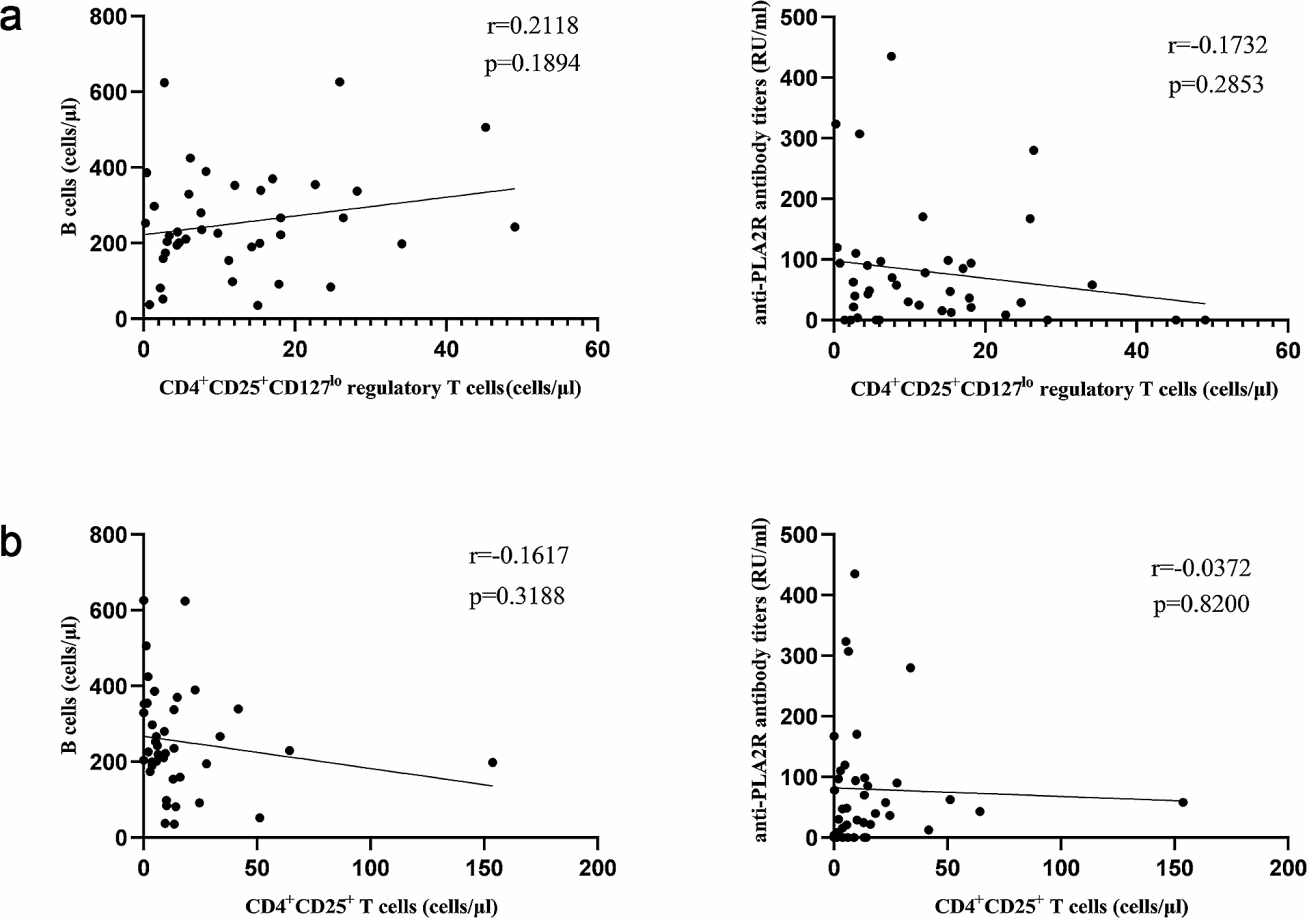
Comparison of changes in T lymphocyte subsets and B lymphocytes before and after RTX treatment

Given that RTX treatment corrected the disorder of T lymphocyte subsets in PMN patients, we wanted to investigate whether this recovery was related to B lymphocyte or anti-PLA2R antibody titres. Pearson correlation analysis was used, and the results showed that changes in Treg cells and CD4^+^CD25^+^ T lymphocytes in the peripheral blood of PMN patients before and after RTX treatment were not correlated with either the B lymphocyte count or the anti-PLA2R antibody titre (Fig. 4). These data suggest a potential independent mechanism of RTX-induced T-cell subset restoration.

## Discussion

Rituximab (RTX) has been used to treat PMN because it can deplete B cells. In this study, we observed a significant increase in B and T lymphocytes in PMN patients compared to healthy individuals. As expected, RTX treatment effectively improved albuminuria in PMN patients within a 6-month period. RTX treatment also resulted in a reduction in the level of the anti-PLA2R antibody and a significant decrease in the of CD3^−^CD19^+^ B-cell count. Although these findings are consistent with the mechanism of action of RTX and are consistent with previous literature reports [21], the clinical results are not always available. The association between CD19^+^ levels and clinical response to rituximab is only sometimes present [22], supporting the concept that the efficacy of rituximab may also be partly due to non-B lymphocytes. In particular, proteinuria improved in patients who did not experience B depletion [23–25].

Additionally, we observed that PMN patients achieved a response rate of 72.5% (partial response + complete response) after 6 months of RTX treatment, for an infection rate of 15.15%. These treatment outcomes are similar to those from previous studies [26]. However, it is important to note that our study followed up with patients for 6 months after treatment, and the recovery of B lymphocyte counts may not have been fully realized during this period. Therefore, long-term follow-up and analysis are necessary to determine the sustained response rate and infection rate after treatment.

In addition, rituximab (RTX) has been proven to potentially affect T lymphocytes in autoimmune diseases. Research on systemic lupus erythematosus showed that effector memory T cells in the CD4^+^ and CD8^+^ T-cell subsets increased after RTX treatment, while a decreasing trend was observed for naïve T cells [27]. A significant increase in the percentage of Tregs among CD4^+^ T cells was observed after six months of RTX treatment in refractory myasthenia gravis [28]. sTIM-3 and sCD25 are regarded as T-cell activation and exhaustion serum markers, respectively, in research on granulomatous lymphocytic interstitial lung disease (GLILD) in CVID patients. Rituximab treatment indirectly caused a trend toward reduced T-cell activation and exhaustion markers sCD25 and sTIM-3 [29].

Notably, our analysis further revealed specific alterations in T-cell subsets within the PMN patients. Specifically, before RTX treatment, the percentages of CD4^+^ T cells, CD4^+^ central memory T cells, and CD4^+^ effector memory T cells and the ratio of CD4^+^ to CD8^+^ T cells were significantly greater in PMN patients than in the normal control group. Conversely, the percentages of CD8^+^ T cells, CD8^+^ central memory T cells, CD8^+^ effector memory T cells, and Treg cells were lower in PMN patients than in healthy controls before RTX. These findings were consistent with previous research [16–17]. In this study, although RTX treatment did not affect the total number of T lymphocytes, CD4^+^ T cells, CD8^+^ T cells, or NK cell counts in the peripheral blood of PMN patients, specific CD4^+^ T-cell subsets, including Treg cells, the CD4^+^/CD8^+^ ratio, and CD4^+^CD25^+^ T cells (IL-2α), were restored significantly after RTX treatment (*P* < 0.05). These findings suggest that RTX effectively inhibite the activation of CD4^+^ T cells and suppress the immune response in PMN patients; they also highlight the potential role of T-cell subsets in the pathogenesis of PMN, indicating an imbalance in autoimmune activation and regulation within this disease.

The proliferation and differentiation of Tregs in the thymus, as well as their peripheral functions, rely heavily on the activation of the IL-2/IL-2R system. IL-2/IL-2R also contributes to maintaining peripheral Treg cell homeostasis [30]. The IL-2Rα chain, also known as CD25, is expressed on the surface of T lymphocytes. In this study, we observed a significant increase in CD4^+^CD25^+^ T lymphocytes but not in IL-2 levels, which also included the CD25 phenotype, among PMN patients. Moreover, RTX treatment noticeably restored the IL-2Rα and Treg cell levels in PMN patients. These findings suggest that both CD4^+^ T-cell activation and Treg cell inhibition coexist in PMN patients. However, the exact mechanism linking IL-2Rα and Treg cells needs to be investigated in future research.

In conclusion, in this study, we showed that PMN patients exhibited an imbalance in T lymphocyte subsets, and this imbalance could be partially recovered after RTX treatment. Additionally, we observed that the RTX-induced changes in peripheral blood CD4^+^ T lymphocyte subsets were not associated with B lymphocyte counts or anti-PLA2R antibody titres. This finding suggests that the effect of RTX on T lymphocyte subsets in PMN patients is independent of B lymphocyte depletion and changes in anti-PLA2R antibody levels, indicating the probable potential impact of RTX on improving cellular immunity.

### Electronic supplementary material

Below is the link to the electronic supplementary material.

Supplementary Material 1

## Data Availability

All data generated or analyzed during this study are included in this article. Further enquiries can be directed to the corresponding author.
