# Supplementary material for: Rituximab may affect T lymphocyte subsets balance in primary membranous nephropathy
Source: BMC Nephrol. 2024 Mar 6;25:86. doi: 10.1186/s12882-024-03521-1 (PMC10918849; doi:10.1186/s12882-024-03521-1)
Supplement: Supplementary file 1 — Supplementary Material 1 [file 12882_2024_3521_MOESM1_ESM.docx]

**Supplementary Table 1:** Comparison of baseline data between the PMN patients and the healthy group

|  | **Healthy Group (n=25)** | **PMN Group (n=58)** | **T/Z** | **P-value** |
| --- | --- | --- | --- | --- |
| **Male, n(%)** | 14(56.00) | 41(71.00) | -1.291 | 0.197 |
| **Age, years** | 47(43.00,49.50) | 53(38.00,65.00) | -1.639 | 0.101 |
| **eGFR, ml/min/1.73m²** | 105.30(98.05, 112.30) | 91.95(74.43, 110.88) | -2.258 | 0.024 |
| **Albumin, g/L** | 46.48±2.31 | 23.38±5.74 | -26.149 | <0.001 |
| **Serum creatinine, μmol/L** | 63.85(52.30, 77.6) | 74(59.25, 93.95) | -2.093 | 0.036 |
| **Cholesterol, mmol/L** | 5.13(4.42, 5.67) | 6.75(5.69, 8.09) | -4.485 | <0.001 |
| **Triglyceride, mmol/L** | 1.63(0.80, 2.08) | 2.28(1.60, 3.24) | -3.189 | 0.001 |

PMN, Primary membranous nephropathy.
